# Could cryopreserved human semen samples be stored at -80°C?

**DOI:** 10.5935/1518-0557.20180016

**Published:** 2018

**Authors:** Carlos R Vaz, Tamara Lamim, Rafael A Salvador, Anna P B Batschauer, Vera Lucia L Amaral, David Til

**Affiliations:** 1 Laboratório de Biotecnologia da Reprodução (LBR), Universidade do Vale do Itajaí (UNIVALI), Itajaí, Santa Catarina, Brazil

**Keywords:** cryopreservation, sperm viability, storage

## Abstract

**Objective:**

To evaluate storage time effects in cryopreserved human semen samples, kept in the freezer at a controlled temperature of -80°C, on sperm viability after thawing.

**Methods:**

We used 20 semen samples. Each sample was cryopreserved in 10 fingers, which were divided into five groups: one group was kept in cryogenic canisters throughout the experiment(control), and four groups were kept in a VIP Ultra Low MDF-U76V- PE freezer, with the temperature set at -80°C, for 24, 48, 72 and 96 hours, respectively. After the exposure time, the samples were stored in cryogenic canisters after being thawed. The analyzed parameters were: motility, vitality and mitochondrial activity.

**Results:**

After thawing, we noticed decreased sperm motility, vitality and mitochondrial activity, when comparing the tested groups with the control group, as well as a progressive reduction in the analyzed parameters between the times evaluated.

**Conclusions:**

Cryopreservation of semen samples at -80°C is potentially harmful to sperm viability, causing damage when submitted to longer exposure times.

## INTRODUCTION

Sperm cryopreservation is a technique developed in the 1950s to maintain structural integrity and cell viability after submission to low temperatures (Bunge *et al*., 1954), maintaining spermatozoa in a state of metabolic arrest, avoiding cell aging and preserving their viability and fertility capacity indefinitely (Gao *et al*., 1997).

The most commonly used method for semen cryopreservation is slow and controlled freezing, which enables the use of adequate cooling rates, reducing the possibility of intracellular crystal formation, as it leads the cells to a uniform dehydration (Gilmore *et al.,* 2000).

After efficacy and safety confirmation of cryopreserved semen use by assisted reproduction techniques, in which similar pregnancy rates to those of non-cryopreserved semen were obtained, semen banks started to be developed (Leibo *et al*., 2002). According to the World Health Organization (WHO, 2010), semen banks serve several purposes and may be used by women with or without male partners who seek semen from anonymous donors and by men undergoing clinical procedures that are harmful or fertility limiting, such as vasectomy, cytotoxic agent treatments or radiotherapy (Chian & Quinn, 2010). Another purpose of semen banks is anonymous donation, where healthy men in their reproductive years seek the altruistic banks to collaborate with those who have difficulty conceiving, donating their semen samples (Daniels, 2006).

Whenever there is a need for transporting cryopreserved human samples, it is carried out in dry ice, where the specimen is maintained at approximately -80°C, or in cryogenic bottles containing liquid nitrogen (-196°C). Dry ice is the most practical means for transporting cryopreserved semen samples, because of temperature variations that can occur in the transport of these samples (Carrell *et al*., 1996; Til *et al*., 2016).

In addition to temperature variations during transport, cryopreservation itself may be detrimental to semen samples. According to Purdy (2006), the cryopreservation process places the spermatozoa in unfavorable conditions, promoting cellular stress, which can cause structural damage and functional changes (Schüffner *et al*., 2008) that alter sperm parameters. Chian & Quinn (2010) demonstrated a motility decrease of 45% after freezing and thawing seminal samples, ranging from 25% to 75%, depending on the initial sample quality (WHO, 2010). Amann & Pickett (1987) found changes in cell membrane structure after cryopreservation, which may lead to loss of sperm function or even, cell death. For these reasons, motility and vitality are the main variables to be evaluated in semen cryopreservation techniques (Cavalcante *et al*., 2006).

Another important factor to be analyzed post-freezing is mitochondrial membrane integrity which is a fundamental factor for sperm physiology, since it is responsible for most of the cell energy production, enabling flagellar movement (Câmara & Guerra, 2008). This organelle has mitochondrial DNA (mtDNA) capable of transcribing several proteins into oxidative phosphorylation; therefore, potential changes in the mitochondrial membrane, or mtDNA mutations, may interfere with sperm characteristics and male fertility (Câmara & Guerra, 2008).

This study aimed to evaluate the influence of cryopreserved seminal samples storage duration, kept at -80°C for up to 96 hours. This data is important to increase transportation reliability, to establish the maximum time of dry ice maintenance without harming semen quality and to guarantee the preservation of patients' samples during the transportation.

## MATERIALS AND METHODS

For performance testing purposes, 20 semen samples were used, from men whose semen were collected when they were being submitted to spermogram in a clinical analysis laboratory located in Itajaí. The normozoospermic samples were cryopreserved using the Yolk Buffer^®^ Test medium (TYB, Irvine Scientific, USA) in a 1:1 ratio. Each donor sample was packed in 10 identified 0.50 ml straws and taken to a refrigerator at 6°C (±2) for 30 minutes, then exposed for 10 minutes to the liquid nitrogen steam with the aid of a (-80°C), and then immersed in liquid nitrogen at -196°C, according to the standard protocol of the Reproduction Biotechnology Laboratory of the University of Vale do Itajaí, adapted from the protocol proposed by REDLARA (2006). 

After cryopreservation, each donor sample (10 straws) was divided into five groups: Group 1 (Control), Group 2 (24 Hours), Group 3 (48 Hours), Group 4 (72 Hours) and Group 5 (96 hours) in duplicate.

Group 1 (Control) straws were kept in liquid nitrogen (-196°C) throughout the experiment, being analyzed after thawing without being exposed to a -80°C temperature, confirming the cryopreservation technique efficacy, and serving as analysis control for dry ice time exposure effects from other groups. Samples from Groups 2 (24 Hours), 3 (48 Hours), 4 (72 Hours) and 5 (96 Hours) were taken from liquid nitrogen and transferred to the VIP Ultra Low MDF-U76V-PE freezer, in a temperature set at -80°C, simulating dry ice storage conditions (adapted from Til *et al.*, 2016).

During the experiment, every 24 hours (24/48/72/96 hours) a group was removed from the freezer and returned to liquid nitrogen, simulating routine restocking in practices.

After returning to cryogenic bottle, the samples were taken from the liquid nitrogen and held for 25 minutes at 37°C; then desiccated in a conical tube containing HTF-HEPES (Life Global^®^), supplemented with 10% Ingámed synthetic serum (INGÁMED^®^) in a 1:2 ratio, centrifuged for 6 minutes at 1500rpm (800G) and resuspended in 400µL of the same medium. After thawing, we analyzed sperm parameters that indicate its viability (motility and vitality) according to the REDLARA (2006), and its mitochondrial activity, as per described by Hrudka (1987). For statistical comparison of results, we performed an analysis of variance (ANOVA) and the Tukey test (significance level of 5%).

This study was approved by the research ethics committee of the University of Vale do Itajaí.

## RESULTS

**Figure 1:**
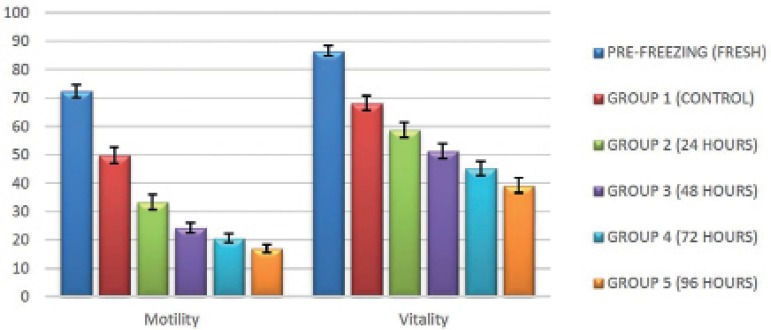
Mean value, in percentage, of the total motility rate and vitality of the samples for the different pre- and post-freezing groups.

When comparing the mean values of motility and vitality, we noticed that all groups suffered a reduction in their post-freeze values, in relation to the fresh sample (Figure 1).

When analyzing the recovery rates of tested groups vis-a-vis the Control Group, we noticed that there was a decrease in motility values (*p*<0.001). However, between the 24 and 48 hour-groups, there was no statistic difference (*p*>0.05), despite a small decrease in values. When analyzing 72 and 96 hour-groups, there was a significant decrease in this parameter, compared to 24-hour Group (*p*<0.001), but there was no difference compared to the 48-hour Group (*p*>0.05) (Table 1), indicating a tendency to stabilize the motility decrease after 48 hours.

**Table 1:** Mean (±standard error), percentage of total motility and vitality recovery rate of different post-freezing group samples.

	Group 1 (Control)	Group 2 (24 hours)	Group 3 (48 hours)	Group 4 (72 hours)	Group 5 (96 hours)
**Motility (%)**	69.48 (±4.05)^a^	47.48 (±4.40)^b^	34.89 (±3.37)^b,c^	29.74 (±3.1)^c^	24.42 (±2.51)^c^
**Vitality (%)**	78.82 (±1.84)^a^	67.91 (±2.81)^b^	59.34 (±2.85)^b,c^	52.25 (±2.72)^c,d^	45.24 (±2.78)^d^

* Different letters in same row differ. *p*<0.05.

Similar results can be found when comparing vitality recovery rates between the control group and the 48, 72 and 96 hour-groups, which presented statistical difference (*p*<0.001). When comparing the Control Group with the 24-hour Group, no difference (*p*>0.05) was found, despite decreases in values. The same results were found when we compared the 24-hour Group to the 48-hour Group (*p*>0.05); the 48-hour Group to the 72-hour Group (*p*>0.05); and the 72-hour Group to the 96-hour Group (*p*>0.05). Despite this, the vitality values from the 72-hour and the 96-hour groups were significantly lower than those from the 24-hour group (*p*<0.01); and the 96-hour group results were significantly lower than those from the 48-hour group (*p*<0.01). This indicates a slow and progressive decrease in sperm cell vitality (Table 1).

This data shows that -80°C storage time is determinant for semen quality. We found that sperm parameters decrease progressively and continuously. The decay may not be significant in short periods of time (every 24 hours); but when compared to longer time intervals, we can find a statistical reduction in these values. This was more significantly in vitality, since for this analysis there are only two variables (stained and non- stained), whereas in motility there are three analyzed variables (progressive, non-progressive and immobile).

Mitochondrial activity showed a decrease in Class I sperm (higher activity) and an increase in Class IV (lower activity) sperm throughout the time periods evaluated (Table 2).

**Table 2:** Mean (±standard error), percentage of I, II, III e IV classes with 3,3’-diaminobenzida staining, for spermatozoa mitochondrial activity of control and tested samples

	Group 1 (Control)	Group 2 (24 hours)	Group 3 (48 hours)	Group 4 (72 hours)	Group 5 (96 hours)
**Class I**	58.8 (±3.12)^a^	45.85 (±2.47)^b^	44.50 (±2.23)^b,c^	42.75 (±2.27)^b,c^	35.00 (±2.36)^c^
**Class II**	17.95 (±1.98)	21.60 (±2.18)	20.25 (±1.95)	20.00 (±1.86)	23.00 (±1.94)
**Class III**	12.70(±1.14)	18.95 (±1.16)	15.75 (±1.14)	16.75 (±1.11)	13.00 (±1.23)
**Class IV**	10.55 (±1.57)^a^	13.60 (±1.24)^a^	19.50 (±1.19)^b^	20.50 (±1.45)^b,c^	29.00 (±1.43)^c^

* Different letters in same row differ. *p*<0.05.

## DISCUSSION

There was motility and vitality decrease between fresh and thawed samples. This may be explained by the chemical and physical stress experienced by sperm cells during cryopreservation, such as cell dehydration, recrystallization, cellular functional alterations and membrane structural damages (Schüffner *et al*., 2008; Petrunkina, 2007). According to Agarwal (2000), sperm motility and vitality rates may decrease by 25-75% after cryopreservation. In this study, maintenance of motility at a range of 38% to 100% and vitality at 65% to 97% after cryopreservation, among donor samples, indicated that each individual may have a different response to cryopreservation. Similar results were reported by Til *et al*. (2016), who observed reduction rates of 19% to 94% in motility and from 27% to 80% in vitality.

Comparison between the groups exposed to -80°C and the control group, showed motility and vitality reduction in all the time periods tested, suggesting that this storage temperature is detrimental to cryopreserved semen samples. Trummer *et al*. (1998) found similar results in their studies, in which after 7 days of storage at -70°C, mean spermatic motility showed a decline of 47%.

These drops may be due to spermatic membrane damage, due to temperature variations to which these samples were exposed. Karow (1974) explains that recrystallization occurs at temperatures close to -87°C, and this enables the formation of intracellular ice crystals, these being the major cause of damage to cell viability. The samples tested in this study went through this critical point during storage, in addition to being kept at a temperature very close to this value, which may have caused cellular damage due to sperm cell recrystallization, causing drops in sperm parameters. 

Another hypothesis for divergences between vitality and motility values is the change in membrane permeability, caused by the stress that samples were submitted to in this temperature variation (Benson *et al*., 2012), because membrane permeability is evaluated by vitality test, not being detected in motility tests. However, both analyzed parameters showed a continuous decrease with time, indicating that the damage suffered by sperm cells should not be related solely to intracellular crystal formation, occurring during storage temperature variation, but that there are other factors acting continuously throughout the sample exposure to-80°C. 

At temperatures below -70°C, Brotherton (1990) reports that cell aging-related enzymes become virtually inactive, keeping cells in a latency state. Despite this experiment, to maintain samples in a controlled environment at -80°C with a variation of ± 1°C, they were stored in groups, inside a packaging destined for transport, hypothesizing that the samples' actual temperatures were higher than the storage environment temperature (-80°C), approaching the temperature suggested by Brotherton (1990), thus enabling the activation of these enzymes, removing cells from the latency state and reactivating their function.

After enzymatic reactivation, the decline in sperm energy metabolism (motility, vitality) may be associated with high concentrations of reactive oxygen species (ROS) in the semen (Baumber *et al*., 2002). Among reactive oxygen species that may be present in the semen, hydrogen peroxide (H_2_O_2_) is cited by Armstrong *et al*. (1999) as one of the main factors responsible for motility and energy production (ATP) inhibition due to its toxicity to human spermatozoa. Maia (2003) concluded in his study that semen manipulation, such as the one occurring during cryopreservation, can lead to a reduction of spermatozoa antioxidant defenses, causing sperm values to decrease.

Paiva (2010), in his study, incubated spermatozoa at 37°C, revealing statistically significant differences after 24 hours, showing a continuous decrease in sperm viability, by 96 hours. The same was found in the present study, suggesting that -80°C is not able to keep sperm cells in the latency state obtained by the cryopreservation process, since the continuous drop in motility and vitality values resembles those observed by Paiva (2010), when evaluating sperm survival time. Til *et al.* (2016) found similar results when evaluating the dry ice (-79°C) transport of cryopreserved samples, proving the ineffectiveness of this temperature in maintaining semen viability.

Motility decrease may also be correlated to mitochondrial damage, which can be seen through the mitochondrial activity test. These analyses' results show a decrease in class I spermatozoa (greater mitochondrial activity) and an increase of class IV spermatozoa (less mitochondrial activity), showing that time exposure to -80°C temperature impairs mitochondria spermatozoa action, contributing to a decrease in analyzed sperm values.

The results show that the storage of semen samples at a -80°C temperature is potentially detrimental to sperm quality, even in the shortest period evaluated (24 hours), emphasizing the need to find other means of transport for these cells, which allow temperatures maintenance closer to that of liquid nitrogen. However, even with a large decline in sperm quality, samples maintained at -80°C during transport can be considered effective when the assisted reproduction technique of choice is intracytoplasmic sperm injection (ICSI), since, as described by Alper (2012), the procedure requires only a single viable sperm to be performed.

## CONCLUSION

The maintenance of seminal samples at -80°C is potentially harmful to spermatozoa, failing to maintain cell viability, removing cells from their latency state, leading to a progressive drop in sperm parameters. This decrease becomes more apparent over time, negatively affecting motility, vitality and mitochondrial activity of the samples. Other methods of storage during transport, with the capacity to maintain cryopreserved samples, should be sought at temperatures closer to that of liquid nitrogen.
